# Implementation of a Surveillance System for Severe Acute Respiratory Infections at a Tertiary Care Hospital in Austria: Protocol for a Retrospective Longitudinal Feasibility Study

**DOI:** 10.2196/47547

**Published:** 2023-08-03

**Authors:** Ziad El-Khatib, Lukas Richter, Andreas Reich, Bernhard Benka, Ojan Assadian

**Affiliations:** 1 Austrian Agency for Health and Food Safety Vienna Austria; 2 Landesklinikum Wiener Neustadt Wiener Neustadt Austria

**Keywords:** severe acute respiratory infection, SARI, Austria, influenza, European Union, COVID-19, respiratory, data retrieval, information retrieval, electronic health record, EHR, health records, health record, surveillance, risk, database structure, incidence, data collection

## Abstract

**Background:**

The risk of a large number of severe acute respiratory infection (SARI) cases emerging is a global concern. SARI can overwhelm the health care capacity and cause several deaths. Therefore, the Austrian Agency for Health and Food Safety will explore the feasibility of implementing an automatic electronically based SARI surveillance system at a tertiary care hospital in Austria as part of the hospital network, initiated by the European Centre for Disease Prevention and Control.

**Objective:**

We aim to investigate the availability of routinely collected health record data pertaining to respiratory infections and the optimal approach to use such available data for systematic surveillance of SARI in a real-world setting, describe the characteristics of patients with SARI before and after the beginning of the COVID-19 pandemic, and investigate the feasibility of identifying the risk factors for a severe outcome (intensive care unit admission or death) in patients with SARI.

**Methods:**

We will test the feasibility of a surveillance system, as part of a large European network, at a tertiary care hospital in the province of Lower Austria (called Regional Hospital Wiener Neustadt). It will be a cross-sectional study for the inventory of the electronic data records and implementation of automatic data retrieval for the period of January 2019 through the end of December 2022. The analysis will include an exploration of the database structure, descriptive analysis of the general characteristics of the patients with SARI, estimation of the SARI incidence rate, and assessment of the risk factors and different levels of severity of patients with SARI using logistic regression analysis.

**Results:**

This will be the first study to assess the feasibility of SARI surveillance at a large 800-bed tertiary care hospital in Austria. It will provide a general overview of the potential for establishing a hospital-based surveillance system for SARI. In addition, if successful, the electronic surveillance will be able to improve the response to early warning signs of new SARI, which will better inform policy makers in strengthening the surveillance system.

**Conclusions:**

The findings will support the expansion of the SARI hospital-based surveillance system to other hospitals in Austria. This network will be of use to Austria in preparing for future pandemics.

**International Registered Report Identifier (IRRID):**

PRR1-10.2196/47547

## Introduction

Globally, there has been an increase in acute respiratory infections, which are considered among the major causes of morbidity and mortality across all age groups [1]. Annually, over 4 million people die from acute respiratory infections, and the majority are due to lower respiratory tract infections [1]. Mortality is high in infants, children, and older people, especially in low- and middle-income countries [2]. Therefore, public health surveillance is an essential tool for monitoring population-level changes in the incidence and burden of respiratory infectious diseases, which is essential for their prevention and targeted control [3].

The COVID-19 pandemic has shown the importance of routine syndromic surveillance of respiratory infections, especially new cases of severe acute respiratory infection (SARI) [4,5]. Currently, there is a need to invest more into systematic data collection on SARI, including the identification of risk factors associated with the SARI cases, which is going to guide policy makers in tailoring appropriate interventions to reduce the SARI public health burden.

Currently, a total of 27 countries in the European Union and 3 countries in the European Economic Area are part of a SARI surveillance network, in collaboration with the European Centre for Disease Prevention and Control (ECDC) and the World Health Organization [6]. The countries using the SARI surveillance network report to the ECDC about the population under their surveillance. This includes persons of all ages living in the catchment areas of the hospitals participating in the national surveillance systems (also, the target population tends to be representative of the general population) [6].

There is concern about the acute emergence of a large number of severe infectious cases, which can overwhelm the health care capacity and cause several deaths. Therefore, in 2022, the ECDC took the initiative to establish a hospital network sustainable infrastructure to conduct enhanced surveillance of SARI, including the performance of studies on the impact and burden of diseases. Thus, it is becoming important and crucial to have at the EU level a hospital-based surveillance system for the monitoring of incidence trends and an early detection system for epidemics, including the identification of the risk groups for severe diseases to measure the impact of these diseases [7].

Austria is participating in this network and is initiating such SARI hospital-based surveillance for the first time. In this feasibility study, we aim to investigate the availability of routinely collected inpatient health record data pertaining to respiratory infections and the optimal approach to use such data for a systematic automated surveillance of SARI. Furthermore, we aim to describe the characteristics of patients with SARI before and after the beginning of the COVID-19 pandemic. Additionally, we investigate the feasibility of identifying risk factors for a severe outcome (intensive care unit [ICU] admission or death) in patients with SARI at a tertiary care hospital in Austria.

## Methods

### Study Design and Setting

This will be an investigative feasibility and cross-sectional study at an 800-bed tertiary care hospital (Regional Hospital Wiener Neustadt) in the federal state of Lower Austria. The analysis will include the inventory of electronic data records and the implementation of automatic data retrieval between January 2019 and December 2022.

For inclusion, we will include all cases that meet the SARI case definition based on the ECDC surveillance definition; we will also include all patients regardless of their address of residence (ie, either inside or outside Austria). Patients not meeting the SARI case definition will be excluded.

### Setting

#### The Federal State of Lower Austria

The Regional Hospital Wiener Neustadt is located in the federal state of Lower Austria, which is the largest federal state in Austria (area size of 19,174 km^2^ and a population of over 1.6 million) [8]. The health care system in Austria is financed through health insurance subsidies provided to hospitals at the federal, regional, and municipal levels [8]. Lower Austria is the only Austrian federal state where all hospitals are legal entities of one single health care provider, the Niederösterreichische Landesgesundheitsagentur (NÖ-LGA), which offers clinical health care services, from basic to specialist, including the handling of the operative management of the hospitals.

Lower Austria is located in the northeast of Austria, where its borders are adjacent to two neighboring countries (Czech Republic and Slovakia). Since 2008, the federal state of Lower Austria has initiated six cross-border health care projects. Additionally, in 2017, The Lower Austrian Health and Social Fund launched a so-called “Initiative Health Across,” which combines all cross-border health care activities in Lower Austria. The aim of this project is to facilitate optimum and equitable usability of the health care services for the population living in the border areas through close cooperation of the health service providers in a simple and uncomplicated way [8].

#### Hospital

The Regional Hospital Wiener Neustadt is an 804-bed tertiary care facility located 60 km south of the Austrian capital Vienna. The hospital provides all major fields of medicine and surgery to large parts of eastern Austria, including the border regions near Slovakia and Hungary, and in Styria to the south. The Regional Hospital Wiener Neustadt is an acute and specialist tertiary referral center with 21 anesthesiology ICU beds, 14 medical intermediate care unit beds, and 21 stroke unit beds, which cares for approximately 35,000 inpatients and 390,000 outpatients, and performs more than 21,000 surgical procedures annually. The medical and nursing staff of the hospital cares for both pediatric and adult patients with respiratory tract infection.

#### Austrian Agency for Health and Food Safety

The Austrian Agency for Health and Food Safety (AGES) is a government-owned agency attached to the Austrian Federal Ministry of Social Affairs, Health, Care and Consumer Protection and the Austrian Federal Ministry of Agriculture, Regions and Tourism. AGES executes several federal tasks, including public health issues and research in the areas of infectious diseases (in addition to other areas, eg, agriculture, food and feed safety, veterinary medicine, radiation protection, medical devices, and pharmaceuticals). In Austria, certain infectious diseases fall under the category of mandatory reporting. Therefore, these diseases are registered in a national registry called the Epidemiological Reporting System (EMS), which is used by laboratories located at the hospitals. The EMS has a central national registry, which is hosted at the Austrian Federal Ministry for Social Affairs, Health, Care and Consumer Protection and maintained under the Institute of Surveillance and Infectious Diseases Epidemiology at AGES [9].

### Data Sources and Variables: Hospital Data Management System

We will be using the data from the EMS together with medical data stored in the local hospital information system. We will include the following information from the clinical records about the patients: the *International Statistical Classification of Diseases, Tenth Revision* (*ICD-10*) code; district of residency; gender (man/woman); date of birth; SARI date onset; bronchoalveolar lavage; tissue from biopsy or autopsy, other, or unknown/not specified; clinical symptoms; existing underlying conditions (yes/no); asthma (yes/no); diabetes (yes/no); cancer (yes/no); immunocompromised (yes/no); heart (yes/no); kidney (yes/no); lung (yes/no); liver (yes/no); neurological (yes/no); obesity (no, BMI 30-40, BMI>40, clinically obese); pregnancy (yes/no); pregnancy trimester (first trimester, second trimester, third trimester); vaccination status related to SARI organisms (yes/no); antiviral prophylaxis (yes/no); antiviral treatment (yes/no); respiratory support (no, oxygen, ventilation, extracorporeal membrane oxygenation [ECMO]); pneumonia diagnosis (no; yes, clinical/abnormal chest x-ray or raised C-reactive protein level), tuberculosis (no history of tuberculosis, history of tuberculosis, positive test during hospital visit); influenza test (not performed, polymerase chain reaction, culture, immunofluorescent assay, other—not specified—test); test result (negative/positive); influenza type (A/B); influenza A subtype (A[H1N1]pdm09, A[H3N2]); ICU admission; outcome (discharged alive/death); and cause of death (not influenza, influenza as a primary cause, influenza as a secondary cause). Only cases with available data will be included in the final analysis, and we will report the proportion of variables with missing data.

### Ethical Considerations

Ethical clearance will be obtained from the Ethics Committee of Lower Austria. A waiver will be requested pertaining to informed consent for this retrospective study. All data will be anonymized to ensure the privacy and confidentiality of patients’ personal information, with each participant assigned a unique identifier.

No compensation is given to the participants.

### Statistical Analysis

The statistical analysis will be conducted, pending the feasibility of the data quality, through the following four steps. In step 1, we will assess the existing database structure, including the exploration of the optimal methods for data retrieval. In step 2, we will conduct a descriptive analysis of the general characteristics of the patients with SARI. The percentage will be calculated by using the total number of patients identified with an underlying condition divided by the total number of patients included in the data set. In step 3, we will calculate the estimation of the SARI incidence rate by using the available data. In step 4, we will then assess the association between the risk factors and different levels of severity of patients with SARI (only cases with available data will be included in the final analysis).

The outcome will be defined as follows: patients with SARI that were discharged alive and not admitted to the ICU, patients with SARI that were discharged alive post admission to the ICU, patients with SARI that died, and patients with SARI with any severe outcome (eg, intensive care, respiratory support, ECMO). We will conduct a logistic regression analysis (backward method) to identify the risk factors associated with a severe outcome. All variables showing an association with a severe outcome at a statistical significance level of *P*<.15 will be included in the multivariable logistic regression analysis. The final variables with a statistical significance of *P*<.05 will be defined as the significant variables. R software (version 4.2.1; R Foundation for Statistical Computing) will be used for the statistical analysis [10].

## Results

The main purpose and objective of this study will be to assess the feasibility of the SARI surveillance system at one of the largest tertiary care hospitals in Austria. The SARI case definition will be defined based on the ECDC SARI protocol. The sources of the SARI surveillance data will include the following: new SARI cases during the calendar week, new SARI cases in which specimens were collected during the reported week, the total number of new hospital admissions to wards under the SARI case definition, the list of *ICD-10* that were used with the number of cases per *ICD-10*, and other specimen and epidemiological data.

## Discussion

As part of a first-time established European SARI hospital-based surveillance network, we will evaluate the feasibility at one of the largest tertiary care hospitals in Austria. We will explore the existing tools to gather data and evaluate the data used in the surveillance reporting and monitoring of SARI, including COVID-19 cases and influenza A. The importance of routine syndromic surveillance of respiratory infections, especially new cases of SARI, became of high importance due to the lessons learned during the COVID-19 pandemic [4,5]. We have conducted a review of the literature and identified that there are no universally agreed standards on the topic of SARI surveillance (El-Khatib Z, unpublished data, June 1, 2023). Therefore, this study and the newly established European network will be useful for the improvement of the preparedness for upcoming pandemics, so we can recommend directions for the future evaluation of SARI surveillance systems.

As shown by the COVID-19 pandemic, early warning and access to relevant data is imperative to act appropriately to ensure the safety of the public from infectious hazards. This study will foster the alertness of public health agencies regarding future respiratory health threats. Therefore, we will be able to improve the response to early warning signs of new SARI signs and eventually better inform policy makers on strengthening the surveillance system. Finally, we should share a potential limitation of this study. The findings of this hospital may lack representativeness at the national level. We may identify additional limitations, pending the data quality, but we could not comment on it at this stage, as the data collection has not started.

The findings of this study will support expansion to other hospitals in Austria and including them in the SARI hospital-based surveillance system. Eventually, such a network will be of use to Austria in preparing for future pandemics.

## Abbreviations

AGES: Austrian Agency for Health and Food Safety
ECDC: European Centre for Disease Prevention and Control
ECMO: extracorporeal membrane oxygenation
EMS: Epidemiological Reporting System
ICD-10: *International Statistical Classification of Diseases, Tenth Revision*
ICU: intensive care unit
NÖ-LGA: Niederösterreichische Landesgesundheitsagentur
SARI: severe acute respiratory infection

## Data Availability

Data sharing is not applicable to this paper, as no data sets were generated or analyzed during this study.
